# Validation of the Catherine Bergego Scale in patients with unilateral spatial neglect after stroke

**DOI:** 10.1590/1980-57642018dn13-010009

**Published:** 2019

**Authors:** Carlos Leonardo Sacomani Marques, Juli Thomaz de Souza, Maicon Gabriel Gonçalves, Taís Regina da Silva, Rafael Dalle Molle da Costa, Gabriel Pinheiro Modolo, José Eduardo Corrente, Rodrigo Bazan, Gustavo José Luvizutto

**Affiliations:** 1 Neurology Department, Botucatu Medical School, UNESP, Botucatu, SP, Brazil.; 2 Department of Rehabilitation, Botucatu Medical School, UNESP, Botucatu, SP, Brazil.; 3 Department of Statistics, Botucatu Medical School, UNESP, Botucatu, SP, Brazil.; 4 Department of Applied Physical Therapy, UFTM - Federal University of Triângulo Mineiro, Uberaba, Brazil.

**Keywords:** unilateral spatial neglect, Catherine Bergego Scale, scale, validation, Portuguese, negligência espacial unilateral, Escala de Catherine Bergego, escala, validação, Português

## Abstract

The Catherine Bergego Scale (CBS) is a scale to evaluates the impact of unilateral spatial neglect (USN) on everyday life of patients after stroke.

**Objective:**

The aim of this study was to evaluate the reliability and comprehension of the Portuguese version of the CBS for patients with USN after stroke.

**Methods:**

This was a cross-sectional study in patients with stroke and USN. The CBS was translated, culturally adapted and applied by two independent investigators. The patients were also evaluated by the Behavioural Inattention Test (BIT), NIHSS, mRS and Barthel scale to assess USN severity, neurological function, disability and autonomy consecutively. Consistency and coherence were analysed using Cronbach’s α, inter-observer reliability by Kappa, and the correlation between the CBS, BIT, NIHSS, mRS, and Barthel was determined using Pearson correlation.

**Results:**

Twenty-two patients were evaluated and the observed Cronbach’s α=0.913. For intra-observer reproducibility, the 10 items showed a reasonable and high reliability between evaluators. The CBS showed a negative correlation with the BIT. There was a low correlation between the BIT and NIHSS, mRS and Barthel index.

**Conclusion:**

The CBS is an adequate and validated scale for assessing patients with USN after stroke in a Brazilian population.

## RESUMO.

A Escala de Catherine Bergego (CBS) é uma escala para avaliar o impacto da negligência espacial unilateral (USN) no cotidiano de pacientes após AVC.

**Objetivo:**

Não há validação da CBS para população brasileira e o objetivo deste estudo foi avaliar a confiabilidade e compreensão da versão em português da CBS para pacientes com USN após AVC.

**Métodos:**

Trata-se de um estudo transversal em pacientes com AVC e USN. A CBS foi traduzida, adaptada culturalmente e aplicada por dois investigadores independentes. Os pacientes também foram avaliados pelo Behaviour Innatention Test (BIT), NIHSS, mRS e Barthel para avaliar a gravidade da USN, função neurológica, incapacidade e autonomia consecutivamente. A consistência e a coerência foram analisadas pelo coeficiente α de Cronbach, a confiabilidade interobservador foi avaliada pelo coeficiente kappa e a correlação entre o CBS, BIT, NIHSS, mRS e Barthel foi avaliada pela correlação de Pearson.

**Resultados:**

Vinte e dois pacientes foram avaliados e observaram-se consistência e coerência pelo coeficiente α de Cronbach=0,913. Na análise da reprodutibilidade intraobservador, os 10 itens da escala apresentaram confiabilidade razoável e alta entre os avaliadores. O CBS mostrou correlação negativa com o BIT. Houve baixa correlação entre BIT e NIHSS, mRS e índice de Barthel.

**Conclusão:**

A CBS é uma escala adequada e validada para avaliar pacientes com USN após AVC em uma população brasileira e de língua portuguesa.

Unilateral spatial neglect (USN) is characterized by the inability to respond to people or objects that are presented on the side contralateral to the lesioned brain, where this symptom cannot be accounted for by either primary motor or sensory deficits. USN is more common in the right-hemisphere, because this region is responsible for double interpretation of space and plays a fundamental role in the process of interpreting sensory stimuli.^1^^-^ 2 This condition is very common after stroke and can contribute to disability, with the incidence of USN varying widely from 10-82%.^3^^-^ 5 The areas of the brain often involved in USN are related to the right hemisphere, such as lesions in the right posterior parietal lobe,^6^^-^ 8 and individuals with USN after stroke present with major functional disabilities, as well as low rates of adherence to rehabilitation programmes.^9^^,^ 10

The gold standard test for grading USN severity is a pen-and-paper test that includes line cancellation, star cancellation, and line bisection tasks. However, this test does not assess functional tasks or activities of daily living (ADLs) in these individuals. Since this instrument is deficient in relating USN to the functioning of these individuals after stroke, the Catherine Bergego Scale (CBS) was devised. The scale analyses the functioning of USN patients for 10 everyday situations after stroke, with the objective of assessing the impact of USN on ADLs.^11^^,^ 12

The CBS has good inter-rater reproducibility, as it is more sensitive than pen-and-paper tests for USN detection. In a study of 18 patients with a diagnosis of USN, the correlation coefficient ranged from 0.59 to 0.99 among the 10 items on the scale. The CBS tool is used in major clinical studies involving USN after stroke to monitor changes in patient behaviour after specific interventions.^13^

The CBS scale is easily and quickly applied by healthcare professionals, as well as being useful for assessing the consequences of USN on ADLs of the patient, allowing for both the adaptation of rehabilitation to individual patient difficulties and the monitoring of recovery. Future research should explore those psychometric properties of the scale not yet evaluated, such as test-retest reliability. Cross-cultural validation in different countries and languages should also be carried out. The CBS can be used in rehabilitation or therapeutic trials to evaluate the functional abilities and gains of patients during rehabilitation.^14^ The CBS has not yet been validated in the Brazilian population and, because it is currently the only instrument that measures the impact of USN on activities of daily living, we carried out this study.

This study aims to evaluate the reliability, comprehension and acceptance of the Portuguese version of the CBS scale in patients with a clinical diagnosis of USN after stroke and to relate the CBS values to the patients’ neurological damage severity, functional status, and autonomy.

## METHODS

### Participants, setting and design

This is a cross-sectional study conducted from June 2017 to March 2018, with patients identified after having experienced an ischaemic stroke who were received at the Neurorehabilitation Department of the Botucatu Medical School. The study was approved by the Ethics and Research Committee (CEP) of the same institution (Protocol 013502/2017). All patients had diagnostic confirmation of stroke with right-hemisphere brain lesion observed on computed tomography and/or magnetic resonance imaging.

### Inclusion criteria

Patients of both sexes, older than 18 years, up to 3 months after discharge from the stroke unit, with a clinical diagnosis of USN, and confirmed by the Behavioural Inattention Test (BIT) as having a score of <129 points were included. All included patients were attending a rehabilitation program at the Botucatu UNESP Rehabilitation Centre 2 times a week since hospital discharge, and all followed the same care protocol.

### Exclusion criteria

Individuals with prior stroke, pre-existing dementia, global aphasia, previous visual disturbances or other associated neurological diseases were excluded.

### Variables and measurements

All patients were evaluated using the following instruments:

- The Behavioural Inattention Test (BIT): The gold standard for clinical diagnosis of USN that consists of six conventional subtests yielding a maximum total score of 146 points, and nine behavioural subtests evaluating aspects of daily life, with a maximum total of 81 points. For a patient to be considered as exhibiting USN, the value of the conventional BIT should be less than 129, and lower rating values indicate worse USN.^2^
- The Catherine Bergego Scale (CBS): Analyses the functioning of the USN patient in 10 everyday life situations. Each question is scored from zero to three, where 0 corresponds to the absence of USN for the given task; 1 corresponds to discrete unilateral space negligence, characterized by slight asymmetry in space exploration; and 3 corresponds to severe unilateral spatial neglect.^12^^,^ 13

After USN diagnoses, the patients were evaluated by the National Institutes of Health Stroke Scale for stroke severity, the modified Rankin Scale (mRS) for functional status and the Barthel index to assess autonomy.

Initially, 10 patients were evaluated by two experienced physiotherapists (Evaluator 1 and Evaluator 2) to identify consistency and coherence by calculating Cronbach’s α coefficient for the adaptations of the necessary CBS items. Subsequently, a total of 22 patients were included to evaluate reproducibility by comparing the results on the items between the two evaluators using the Kappa coefficient.

### Translation and cross-cultural adaptation

- Stage 1. Translation from French to Portuguese: The CBS was initially translated into Portuguese by a certified translator in French.
- Stage 2. Transcultural appropriateness and judges’ assessment: The Portuguese version was sent to a group of 6 judges, consisting of University professors with experience in stroke and rehabilitation, who performed cross-cultural adaptation of the instrument to facilitate ease and consistency of interpretation of the items.
- Stage 3. Back translation from Portuguese to French: After the initial translation, the scale was sent to a professional sworn translator in French for back translation.
- Stage 4. Comparison of the two versions of the scale (scale after translation and reverse translation): Versions 1 (translation into Portuguese) and 2 (the back translation) were evaluated by the authors, comparing them with the original text to correct discrepancies and address them to create a compatible version without inconsistencies.
- Stage 5. Final scale version (Appendix 1): After the transcultural adaptation of the instrument, the individuals were invited to participate in the application of the adapted scale and signed the informed consent form.

### Evaluation of the reliability of the scale in Portuguese

The instrument was applied by two physiotherapists from the neurorehabilitation team, and each evaluator received a copy of the scoring criteria and the rules for using the scale.

Scale application was done independently, with the researcher blinded to the patient’s neurological changes or the other researcher’s score.

### Statistical analysis

The values ​​of mean, standard deviation, median, minimum and maximum values ​​for quantitative variables (age, weight, and body mass index) and frequencies, and percentages for qualitative variables (demographic and CBS questionnaire) were obtained. Considering the initial data (10 patients), Cronbach’s α coefficient was obtained to evaluate the consistency and coherence of the instrument. The Kappa coefficient for each item was calculated with the data from the two evaluators to verify inter-observer reproducibility. The CBS scores were calculated for both evaluators by adding the results of each item for each patient. These values ​​were compared using Student’s paired *t*-test and for differences between the means calculated by the two evaluators. The Pearson correlation coefficients between the CBS, BIT, NIHSS and Barthel scores were also calculated. All analyses were done using the SigmaPlot software for Windows v12.0 (Systat Software Inc., San Jose, CA, USA). In all tests, a p-value of p<0.05 was considered significant.

## RESULTS

Twenty-two patients with a mean age of 62.7±12.6 years were included. The data in Table 1 shows scores on the scales most often used in patients diagnosed with stroke.

**Table 1:** Descriptive measures of characteristics of sample studied.

Variable	N	Mean	SD	Min	Max	Median
Age (years)	22	62.73	12.63	23.00	85.00	64.00
Weight (kg)	22	68.26	18.75	51.60	90.00	69.50
BMI (kg/m^2^)	22	27.66	4.00	18.50	35.90	27.05
Education (years)	22	4.63	1.94	1.00	7.00	5.00
Time since stroke (days)	22	37.36	6.10	30.00	50.00	38.00
NIHSS admission	22	10.59	5.46	0.00	23.00	11.00
NIHSS current	22	4.77	3.10	0.00	11.00	4.50
mRS	22	1.77	1.34	0.00	4.00	1.50
Barthel index	22	61.59	33.07	15.00	100.00	60.00
BIT	22	80.23	37.50	13.00	125.00	89.00
Evaluator 1 CBS	22	11.41	9.40	0.00	30.00	10.00
Evaluator 2 CBS	22	11.55	9.16	0.00	29.00	12.00

BMI: Body mass index; NIHSS: National Institute of Health Stroke Scale; mRS: modified Rankin scale; BIT: Behavioural Inattention Test; CBS: Catherine Bergego Scale.

After translation by a native Brazilian sworn to translate Portuguese into the French language, the judges made observations of the possible changes to be made as highlighted in the annex. The CBS was then adapted to improve patient understanding at the time of scale application. The scale was applied by both evaluators separately, with no possibility of interference between the two.

After statistical analysis of the 22 patients, the Cronbach’s α coefficient was calculated as α=0.913, which shows excellent consistency. In the analysis of intra-observer reproducibility using the Kappa coefficient, a reasonable result was obtained. The related data are shown in Table 2.

**Table 2:** Kappa coefficient for each question of CBS instrument obtained by two evaluators.

Question	Kappa	P
Question 1 (Grooming)	0.7643	0.8088
Question 2 (Dressing)	0.6736	0.5438
Question 3 (Eating)	0.5401	0.7660
Question 4 (Mouth cleaning)	0.6271	0.5438
Question 5 (Gaze orientation)	0.6440	0.9993
Question 6 (Left limb knowledge)	0.6354	0.6767
Question 7 (Auditory attention)	0.7124	0.6767
Question 8 (Collisions)	0.5138	0.2381
Question 9 (Spatial orientation)	0.5562	0.3208
Question 10 (Finding personal belongings)	0.8683	0.9197

The CBS showed a negative correlation with the BIT, i.e. higher scores on the BIT correlated with lower scores on the CBS. The inter-observer agreement rate did not differ significantly between the scales.

The results for the Pearson correlation coefficient between the CBS, BIT, NIHSS, mRS and the Barthel index are outline in Table 3.

**Table 3:** Pearson correlation of CBS scale scores with NIHSS, BIT, mRS and Barthel.

	NIHSS admission	mRS	Barthel index	NIHSS current	BIT	Evaluator 1	Evaluator 2
**NIHSS admission**	1	-0.24037 0.2812	-0.29128 0.1884	0.67149 0.0006	-0.31804 0.1492	-0.21905 0.3274	-0.22637 0.3111
**mRS**		1	-0.05581 0.8052	-0.17313 0.4410	0.32732 0.1370	0.03413 0.8802	-0.00880 0.9690
**Barthel index**			1	-0.41195 0.0568	0.47041 0.0271	-0.28712 0.1951	-0.32825 0.1358
**NIHSS current**				1	-0.19694 0.3797	0.03276 0.8849	-0.00381 0.9866
**BIT**					1	-0.45891 0.0317	-0.46222 0.0303
**Evaluator 1**						1	0.97804 <.0001
**Evaluator 2**							1

NIHSS: National Institute of Health Stroke Scale; mRS: modified Rankin scale; BIT: Behavioural Inattention Test.

## DISCUSSION

In our study, we aimed to validate the CBS in the Portuguese language to obtain a valuable instrument for evaluating daily life skills and prognosis in patients with USN after stroke, as there is no reported objective scale available in Portuguese for evaluating these parameters in this population.^14^

After application of the CBS, excellent correlation was found demonstrating the consistency and coherence of the instrument. The Kappa coefficient indicated good correlation of inter-observer agreement for the CBS when translated and adapted to Brazilian culture. The CBS was easily applied by professionals and then validated for this population. In an inter-observer reproducibility analysis using the Kappa coefficient, the scale for the 10 items ranged from 0.51 to 0.86, showing reasonable and high reliability between the evaluators.

In a previous psychometric evaluation of the CBS, Azouvi et al. (2003) observed that its items follow a pattern of most to least difficult: dressing (question 2), left limb knowledge (question 6) and collides when moving (question 8), personal belongings (question 10), gaze orientation (question 5), spatial orientation (question 9), eating (question 3), auditory attention (question 7) and grooming (question 1), and mouth cleaning (question 4).^13^ In this study, the questions eating, collides when moving, and spatial orientation had lower agreement, and this may be due to higher subjectivity in the interpretation of these questions and higher variability in responses. The items grooming, auditory attention, and personal belongings presented better agreement because they are more objective and are often the functions initially compromised in these patients. The three questions with poor agreement are related to aspects of neglect, reflecting impairment of automatic orienting of attention toward the extrapersonal space, often difficult to quantify objectively.

There was a low correlation between the scores on the CBS and the BIT in the study population. This result reflects the CBS usefulness for quantifying the functional impact of USN on daily activities and for the prognosis of these patients, whereas the BIT is the gold standard instrument commonly used for diagnosing the disease; therefore, the CBS should not be used as a diagnostic tool in this population.^12^^,^ 13 The CBS and the BIT provided distinct assessments of patients with USN, where the CBS evaluates aspects of perceptual and motor dysfunction separately, quantifying aspects of USN separately, while the BIT evaluates perceptual and spatial cognitive motor systems and is highly recommended for assessing these parameters.^15^

Among the study’s findings was the observation that there was also no good correlation between the other scores on the CBS with the NIHSS, the mRS or the Barthel index. The mRS and the Barthel index do not directly assess the impact of USN on daily activities, but aim to identify the degree of independence and self-care in activities of daily life, in relation to only motor abilities, rather than perceptual ones [16]. The NIHSS quantifies the neurological deficits of the stroke patient based on the evaluation of the level of consciousness, speech, language, visual field perception, somatoagnosia, ocular movement, strength, coordination and sensitivity. There was no correlation of the NIHSS when compared with the CBS, as the NIHSS is more focused on diagnosis in the acute phase and underestimates the severity of right-hemisphere lesions.^16^

Azouvi et al. (2003) affirmed previous findings that the CBS can be used to assess behavioural neglect. Being more sensitive than pencil-and-paper tests, with good psychometric properties, rehabilitation professionals need tools to improve care for patients’ individual difficulties and to adapt and monitor the recovery of these individuals.^13^ We believe that the line cancelation and line bisection tasks are easy and objective measures that can be performed in a bedside setting for diagnostic evaluation, while the CBS is more helpful than the pen-and-paper tests for follow up and rehabilitation purposes, since it allows evaluation of the daily routine of patients with USN.

The main limitations are related to the individual’s understanding of issues involving such a specific topic. USN is a theme little known by the population, which is consequently unable to accurately rate the items. However, the CBS is a direct instrument for evaluating the behaviour of patients with USN after stroke in their daily environment that offers simple and quick application by a professional in a clinical or hospital environment. It is of great relevance to carry out cross-cultural validation in several countries and languages, expanding on and providing increasingly objective tools to healthcare professionals for USN treatment.

Based on the results, the CBS is adequate and was validated for the assessment of patients with USN after stroke in a Brazilian multicultural and Portuguese-speaking population.

## APPENDIX 1 Catherine Bergego scale in Portuguese.

**Table:**

	Atividade	0	1	2	3
1	"Esquecer" de lavar o rosto ou se barbear do lado esquerdo				
2	Dificuldade em ajustar o lado esquerdo da manga da camisa ou barra da calça;				
3	"Esquecer" alimentos localizados na parte esquerda do prato				
4	Esquecer a limpeza do lado esquerdo da boca após a refeição				
5	Dificuldade em direcionar espontaneamente o olhar para a esquerda;				
6	Esquecer de utilizar a parte esquerda do corpo, por exemplo, membro superior não é colocado sobre o apoio de braço da cadeira de rodas; ou pé não é colocado sobre o apoio de pés da cadeira de rodas; "ou se esqueça de usar o braço esquerdo quando precisar."				
7	Dificuldade em prestar atenção a ruídos ou às pessoas que se dirigem ao paciente pelo lado esquerdo;				
8	Colisões com objetos ou pessoas situadas à esquerda, como portas ou móveis (mesmo enquanto andando ou em cadeira de rodas);				
9	Dificuldade de orientar-se para a esquerda em locais com os quais está familiarizado ou dentro do serviço de reabilitação;				
10	Dificuldade em encontrar objetos pessoais situados à esquerda no quarto ou no banheiro.				
	TOTAL				

## References

[1] Freeman E (2001). Unilateral Spatial neglect: new treatment approaches with potencial application to occupational therapy. Am J Occup Ther.

[2] Halligan PW, Burn JP, Marshall JC, Wade DT (1992). Visuo-spatial neglect: qualitative differences and laterality of cerebral lesion. J Neurol Neurosurg Psychiatry.

[3] Halligan PW, Marshall JC (2001). Graphic neglect- more than the sum of the parts. Neuroimage.

[4] Luvizutto GJ, Moliga AF, Rizzatti GRS, Fogaroli MO, Moura E, Nunes HRC (2018). Unilateral spatial neglect in the acute phase of ischemic stroke can predict long-term disability and functional capacity. Clinics.

[5] Heilman DM, Van Den Abell T (1980). Right hemisphere dominance for attention: the mechanism underlying hemispheric asymmetries of inattention (neglect). Neurology.

[6] Kerkhoff G (2001). Spatial hemineglect in humans. Prog Neurobiol.

[7] Kim M, Na DL, Kim GM, Adair JC, Lee KH, Heilman KM (1999). Ipsilesional neglect: behavioural and anatomical features. J Neurol Neurosurg Psychiatr.

[8] Plummer P, Morris ME, Dunai J (2003). Assessment of unilateral neglect. Phys Ther.

[9] Swan L (2001). Unilateral Spatial Neglect. Phys The.

[10] Verdon V, Vuilleumier P (2010). Neuroanatomy of hemispatial neglect and its functional components: a study using voxel-based lesion symptom mapping. Brain.

[11] Azouvi P, Marchal F, Samuel C (1996). Functional consequences and awareness of unilateral neglect: study of an evaluation scale. Neuropsychol Rehabil.

[12] Bergego C, Azouvi P, Samuel C (1995). Validation d'une e´chelle d'e´valuation fonctionnelle de l'he´mine´gligence dans la vie quotidienne: l'e´chelle CB. Ann e´adaptation. Med Phys.

[13] Azouvi P, Olivier Sylvie, Montety Godeleine de, Samuel Christiane, Louis-Dreyfus Anne, Tesio Luigi (2003). Behavioral Assessment of Unilateral Neglect: Study of the Psychometric Properties of the Catherine Bergego Scale. Arch Phys Med Rehabil.

[14] Seron X, Deloche G, Coyette F, Seron X, Deloche G (1989). A retrospective analysis of a single case neglect therapy: a point of theory. Cognitive approaches in neuropsychological rehabilitation.

[15] Goedert Kelly M., Chen Peii, Botticello Amanda, Masmela Jenny R., Adler Uri, Barrett Anna M. (2012). Psychometric Evaluation of Neglect Assessment Reveals Motor Exploratory Predictor of Functional Disability in Acute-Stage Spatial Neglect. Arch Phys Med Rehabil.

[16] Cincura C, Pontes-Neto OM, Neville IS, Mendes HF, Menezes DF, Mariano DC (2009). Validation of the National Institutes of Health Stroke Scale, modified Rankin Scale and Barthel Index in Brazil: the role of cultural adaptation and structured interviewing. Cerebrovasc Dis.

